# *Aedes vittatus* in Spain: current distribution, barcoding characterization and potential role as a vector of human diseases

**DOI:** 10.1186/s13071-018-2879-4

**Published:** 2018-05-18

**Authors:** Alazne Díez-Fernández, Josué Martínez-de la Puente, Santiago Ruiz, Rafael Gutiérrez-López, Ramón Soriguer, Jordi Figuerola

**Affiliations:** 1 Estación Biológica de Doñana (EBD-CSIC), Calle Américo Vespucio 26, E-41092 Seville, Spain; 2 CIBER de Epidemiología y Salud Pública (CIBERESP), Seville, Spain; 3 Servicio de Control de Mosquitos, Diputación de Huelva, Huelva, Spain

**Keywords:** DNA barcoding, *Aedes* mosquitoes, Vector-borne diseases

## Abstract

**Background:**

*Aedes vittatus* is currently found in Africa, Asia and Europe, where it acts as a vector of pathogens causing animal and human diseases (e.g. chikungunya, Zika and dengue). Like other *Aedes* species, *Ae. vittatus* is able to breed in artificial containers. The ECDC has recently highlighted the need for molecular tools (i.e. barcoding characterization) that enable *Aedes* species to be identified in entomological surveys.

**Results:**

We sampled mosquito larvae and adults in southern Spain and used a molecular approach to amplify and sequence a fragment of the cytochrome *c* oxidase subunit 1 gene (barcoding region) of the mosquitoes. The blast comparison of the mosquito sequences isolated from Spain with those deposited in public databases provided a ≥ 99% similarity with sequences for two *Aedes* mosquitoes, *Ae. vittatus* and *Ae. cogilli*, while similarities with other *Aedes* species were ≤ 94%. *Aedes cogilli* is only present in India and there are no records of this species from Europe.

**Conclusions:**

Due to the low genetic differences between *Ae. vittatus* and *Ae. cogilli*, the barcoding region should not be used as the only method for identifying *Ae. vittatus*, especially in areas where both of these *Aedes* species are present. This type of analysis should thus be combined with morphological identification using available keys and/or the characterization of other molecular markers. In addition, further entomological surveys should be conducted in order to identify the fine-scale distribution of this mosquito species in Europe.

## Background

Vector-borne pathogens are a global health concern in which mosquitoes play a central role as vectors of pathogens [1]. In Europe both native and invasive species of *Aedes* mosquitoes are involved in the transmission of pathogens including viruses (e.g. dengue and chikungunya [2]) and parasites (e.g. *Dirofilaria* [3]). Of these mosquitoes, the invasive *Aedes albopictus* has received much attention in recent decades due to its role in the transmission of dengue [4] and chikungunya [5] in Europe. Certain *Aedes* species, including *Ae. albopictus*, are able to breed in artificial containers and it is important to develop accurate identification protocols for differentiating native and invasive *Aedes* species that breed in the same area [6–8]. The identification of mosquito species through the characterization of a fragment of the cytochrome *c* oxidase subunit 1 (*cox*1) gene is a useful tool for monitoring the presence of species [9, 10], above all given the difficulties in identifying mosquitoes in larval stages and the current scarcity of trained taxonomists [11]. However, this method requires a previous genetic characterization of the species [12]. This is an important limitation in the case of *Aedes* mosquitoes as this information is not available for most of the species of this genus that breed in Europe [13], despite their importance in pathogen transmission [14].

The aim of this study was to update the current distribution of *Ae. vittatus* and provide the first genetic characterization of the barcoding region of specimens of this species from Europe. Hitherto, sequences from this species were only available from China [15], India [16] and Kenya [17]. In addition, we review here available information on the potential role of this species in the transmission of virus of public health concern.

## Methods

**Fig. 1:**
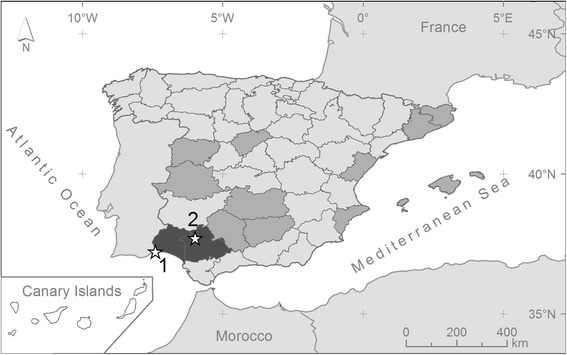
Distribution by provinces of *Ae. vittatus* in Spain. Light grey and dark grey indicates the provinces where the species is absent or present, respectively. The two new records of *Ae. vitattus* reported in this study are marked with stars: 1. Ayamonte (Huelva Province), 2. Castilblanco de los Arroyos (Seville Province)

As a part of an extensive mosquito-monitoring program, a female *Ae. vittatus* was captured in a CDC trap in Ayamonte, Huelva Province (Fig. 1; 37°13'30"N, 7°24'29"W), in June 2015. This sampling site is located in the Guadiana marshes, in the garden of a house close to the built-up area of Ayamonte. At the same time, we also trapped 19 *Ochlerotatus caspius*. In further trapping sessions during 2015 in this area we captured 1145 *Oc. caspius*, 47 *Oc. detritus*, 9 *Cx. pipiens*, 4 *Cx. theileri*, 3 *Cx. perexiguus*, 3 *Culiseta longiareolata* and 2 *Cs. annulata*. Additionally, mosquito larvae were collected from a container in July 2015 in a rural property near Castilblanco de los Arroyos, Seville Province (Fig. 1; 37°41'56"N, 5°58'44"W), in an area characterized by the presence of isolated houses surrounded by scrubland. Larvae were maintained in plastic trays with natural water and fed *ad libitum* with Mikrozell (Hobby Mikrozell 20 ml/22 g) in a climatic chamber at constant conditions (28 °C, 65–70% relative humidity (RH) and 12:12 light:dark photocycle). Adult mosquitoes were fed *ad libitum* with 1% sugar solution. Five to seven days after emergence, adult mosquitoes were anaesthetised with diethyl ether and identified to species level using available taxonomic keys [18, 19] under a stereo-microscope (Nikon SMZ645). The ability of laboratory-reared females to bite humans was checked by exposing the arm of one of the authors (RGL) to mosquito bites. The time elapsed between arm exposure and the beginning of blood-feeding was recorded.

Three mosquitoes (one male and two females) from Seville Province were selected for molecular characterization of the barcoding region and to confirm the morphological identification of the species. A fragment of the right hind-leg of each mosquito was cut-off using a sterile blade and placed on a Petri dish. Genomic DNA was extracted using the Maxwell 16 LEV Blood DNA Kit (Promega, Madison, WI, USA) following the manufacture’s instructions. PCR reactions were performed using the primer pair LCO1490 (5'-GGT CAA CAA ATC ATA AAG ATA TTG G-3') and HCO2198 (5'-TAA ACTT CAG GGT GAC CAA AAA ATC A-3') [20] following Whiteman et al. [21] to amplify a 658 bp fragment of the *cox*1 gene (excluding primers) (see [22]). The presence of amplicons was verified on 1.8% agarose gels. Sequences were resolved in both directions by Macrogen sequencing service (Macrogen Inc., the Netherlands). Sequences were edited using the SequencherTM v4.9 software (Gene Codes Corp., Ann Arbor, MI, USA) and compared with sequences deposited in the GenBank DNA sequence database (National Center for Biotechnology Information) and the Barcode of Life Data Systems (BOLD).

## Results

**Fig. 2:**
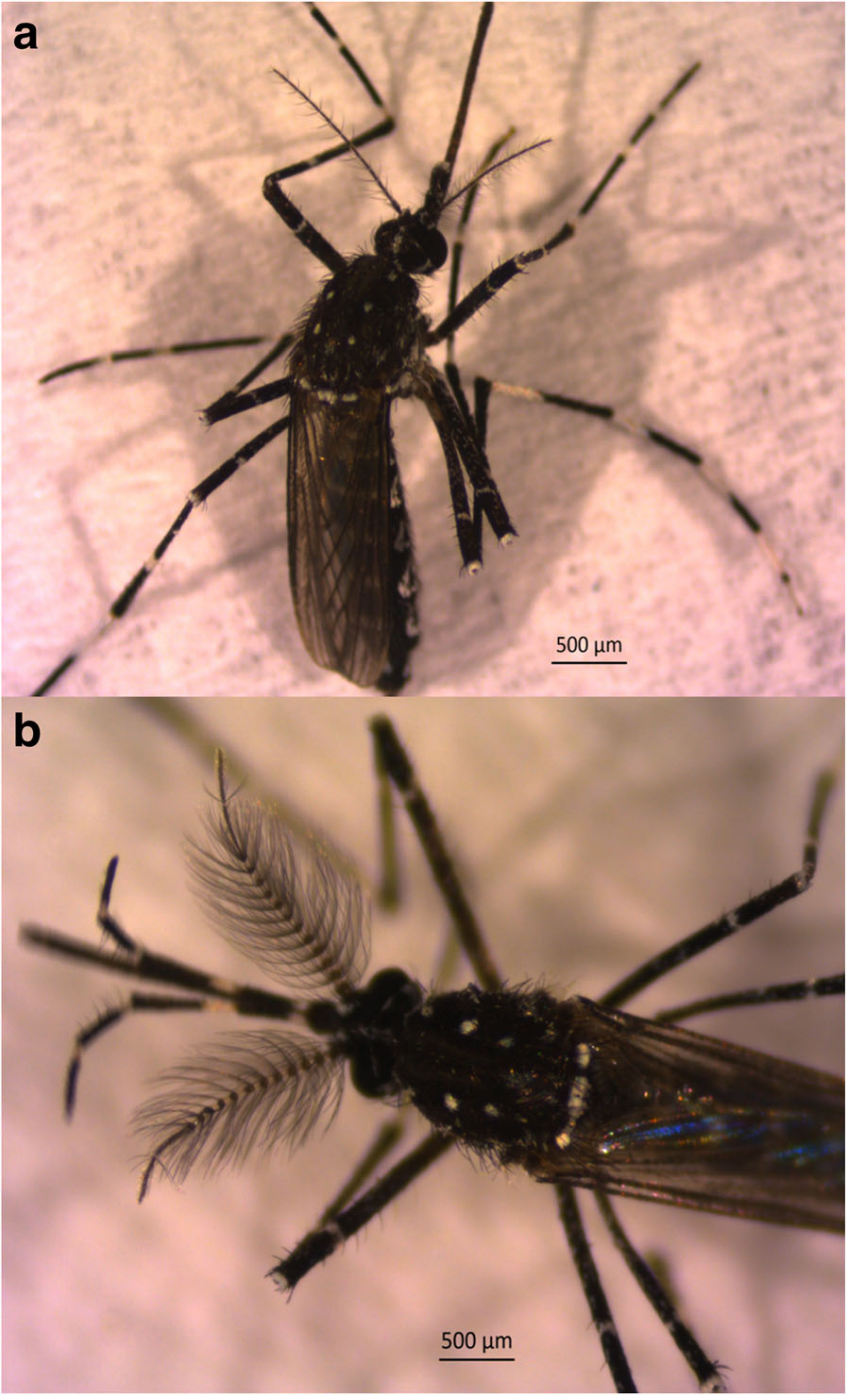
*Aedes vitattus* female (**a**) and male (**b**) captured in the Seville Province

Mosquitoes were morphologically identified as *Ae. vittatus* (Fig. 2). Genetic characterization of the barcoding region of the three mosquitoes provided a unique haplotype. Using the BOLD system, the sequences obtained in our study were identified as *Ae. vittatus* (99.4%) or *Aedes* (*Phagomyia*) *cogilli* (99.0%). Likewise, a 99% overlap between *Ae. vittatus* and *Ae. cogilli* was found using a BLAST comparison with sequences in GenBank, while similarities with other *Aedes* species were ≤ 94%.

The anthropophilic feeding preference of *Ae. vittatus* females was confirmed by the fact that four mosquitoes (57.1%) fed on a human arm after < 5 min of exposure.

## Discussion

We characterized for the first time in Europe the barcoding region of *Ae. vittatus*. A BLAST comparison of this sequence with those deposited in public databases provided a ≥ 99% similarity with sequences of two *Aedes* mosquitoes, *Ae. vittatus* and *Ae. cogilli*. However, *Ae. cogilli*, is only present in India and is not found in Europe [23]. The other *Aedes* sequences on GenBank differed by about 6% from the *Ae. vittatus* sequence isolated here. Although varying between taxa, interspecific differences in the barcoding region are established at 0–2% [24]. Based on the low interspecific differences found between *Ae. vittatus* and *Ae. cogilli*, our results do not support the use of the *cox*1 region as a method for separating these species where they coincide; rather, this method should be combined with morphological identification using available keys or the characterization of other molecular markers. Based on the morphological characteristics of the specimens captured here, we conclude that the mosquitoes we captured belong to the species *Ae. vittatus* [25].

**Fig. 3:**
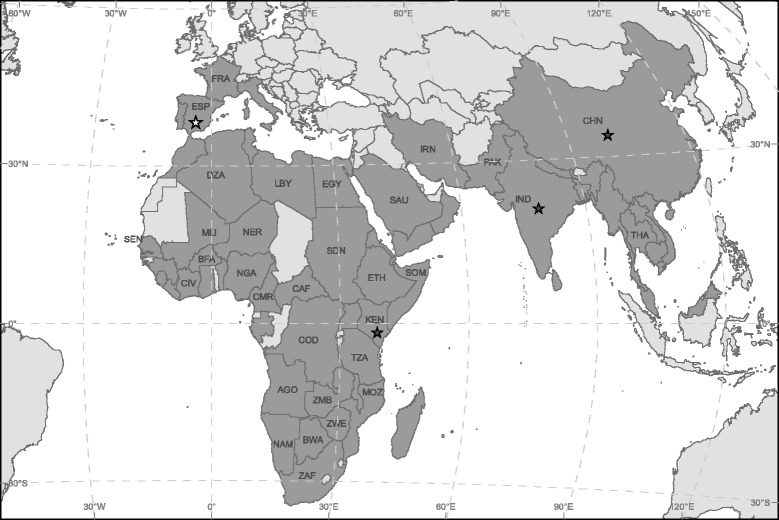
Worldwide distribution of *Ae. vittatus* (dark grey colour). Stars indicate the geographical origin of the previously (black) and new (white) described genetic sequences of the barcoding region

The current distribution of *Ae. vittatus* includes rural and natural areas in Africa, Asia and European countries in the Mediterranean Basin such as France, Italy, Portugal and Spain (Fig. 3). Specifically, *Ae. vitattus* has been recorded with a clear discontinuous distribution from eleven Spanish provinces [26]. Larvae of *Ae. vittatus* have been recorded in a variety of habitats including rock pools, tree holes, domestic containers and hoofprints [27, 28]. In eastern Spain, this species is present in coastal mountainous areas of thermomediterranean and lower mesomediterranean thermotypes [29]. Here, we update the distribution of this species in the Iberian Peninsula and provide the first reports of its presence in the provinces of Huelva and Seville (Fig. 1). In Huelva, an adult female was trapped close to a built-up area, while mosquito larvae belonging to this species were sampled in a rural property in Seville. The mosquito from Huelva was captured in an area close to the town of Ayamonte, which suggests the possibility of contact between this mosquito species and human populations.

The fact that *Ae. vittatus* uses artificial containers for breeding in rural ecosystems may be particularly relevant given its ability to transmit pathogens causing human diseases. In addition to humans, *Ae. vittatus* feed on bovids, sheep/goats and porcupines [30, 31], suggesting its potential role in the transmission cycle of a variety of arboviruses (Table 1). Although *Ae. vittatus* has also been reported to be involved in the transmission of viruses potentially affecting humans, including species of *Alphavirus*, *Flavivirus* and *Bunyavirus* (Table 1), this species probably only has a low risk in Spain. Diagnosis of these diseases and vector surveillance will help elucidate the potential role of *Ae. vittatus* in the transmission of viruses in Europe.

**Table 1:** Main viruses causing diseases transmitted by *Ae. vittatu*s with information of the potential hosts and known distribution of the diseases

Family/Virus	Disease	Hosts	Distribution	Reference
Family Togaviridae (*Alphavirus*)				
Babanki virus	Babanki	Humans, birds	Africa, Europe	[32]
Chikungunya virus	Chikungunya	Humans, birds, domestic animals, monkeys, rodents	Africa, America, Asia, Europe	[33]
Middelburg virus	Middelburg	Humans, domestic animals	Africa	[34]
Semliki Forest virus	Encephalitis	Humans, birds, domestic animals, non-human primates, rodents	Africa, Asia, Europe	[35]
Family Flaviviridae (*Flavivirus*)				
Dengue virus	Dengue	Humans, non-human primates	Africa, South America	[36, 37]
Saboya virus	Saboya	Humans, rodents	Africa	[38]
Wesselsbron virus	Wesselsbron	Humans, domestic animals, monkeys	Africa	[39]
Yellow fever virus	Yellow fever	Humans, non-human primates	Africa, South America	[40, 41]
Zika virus	Zika	Humans, bats, birds, domestic animals, non-human primates	Africa, America, Asia	[42–44]
Family Bunyaviridae (*Bunyavirus*)				
Bunyamwera virus	Bunyamwera	Humans	Africa	[45]

## Conclusions

When identifying *Ae. vittatus* in areas where its distribution overlaps with that of the related Asian species *Ae. cogilli*, the identification of the barcoding region should be combined with morphological identification and/or the characterization of other molecular markers. However, in Europe, molecular tools may allow for the accurate identification of this species due to the great genetic difference (6%) found between Spanish *Ae. vittatus* and other *Aedes* species. Further entomological studies should be conducted in order to identify the fine-scale distribution of *Ae. vittatus* in European countries, where it could play a role in the transmission of viruses with public health relevance.
