# Correction to: *Aedes vittatus* in Spain: current distribution, barcoding characterization and potential role as vectors of human diseases

**DOI:** 10.1186/s13071-019-3737-8

**Published:** 2019-10-14

**Authors:** Alazne Díez-Fernández, Josué Martínez-de la Puente, Santiago Ruiz, Rafael Gutiérrez-López, Ramón Soriguer, Jordi Figuerola

**Affiliations:** 1 Estación Biológica de Doñana (EBD-CSIC), Calle Américo Vespucio 26, 41092 Seville, Spain; 2 CIBER de Epidemiología y Salud Pública (CIBERESP), Seville, Spain; 3 Servicio de Control de Mosquitos, Diputación de Huelva, Huelva, Spain

## Correction to: Parasites Vectors (2018) 11:297 10.1186/s13071-018-2879-4

Unfortunately, the original version of this article [1] contained an error. In the distribution map in Fig. 3, the presence of the mosquito *Aedes vittatus* was incorrectly indicated for Libya and Egypt.

The corrected Fig. 3 is included with this correction.

**Fig. 3:**
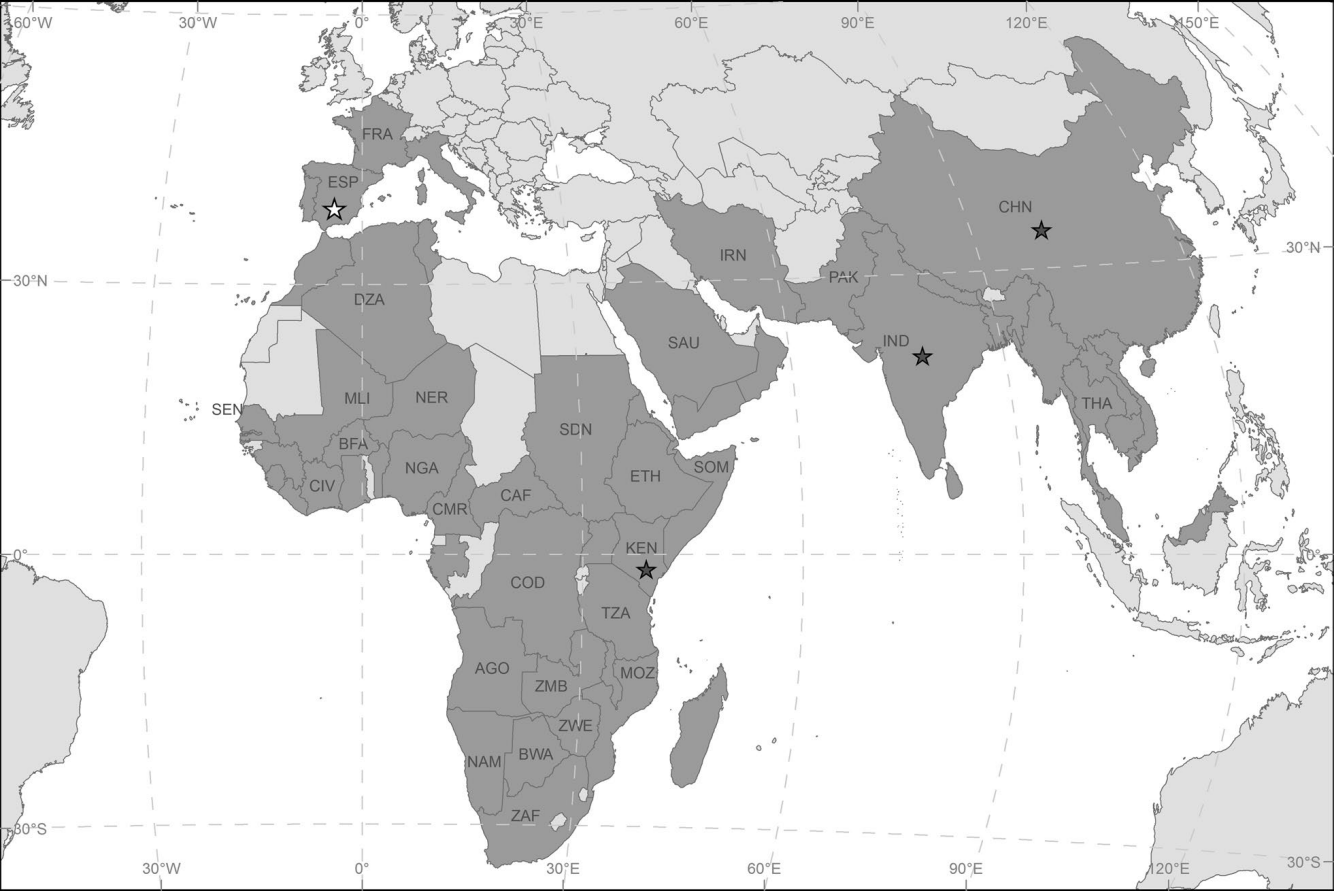
Worldwide distribution of *Ae. vittatus* (dark grey colour). Stars indicate the geographical origin of the previously (black) and new (white) described genetic sequences of the barcoding region

The authors apologize for the inconvenience caused.
